# Transfer hydrogenation catalysis in cells

**DOI:** 10.1039/d0cb00150c

**Published:** 2020-11-05

**Authors:** Samya Banerjee, Peter J. Sadler

**Affiliations:** Department of Chemistry, University of Warwick, Gibbet Hill Road Coventry CV4 7AL UK P.J.Sadler@warwick.ac.uk

## Abstract

Hydrogenation reactions in biology are usually carried out by enzymes with nicotinamide adenine dinucleotide (NAD(P)H) or flavin mononucleotide (FAMH_2_)/flavinadenine dinucleotide (FADH_2_) as cofactors and hydride sources. Industrial scale chemical transfer hydrogenation uses small molecules such as formic acid or alcohols (*e.g.* propanol) as hydride sources and transition metal complexes as catalysts. We focus here on organometallic half-sandwich Ru^II^ and Os^II^ η^6^–arene complexes and Rh^III^ and Ir^III^ η^5^–Cp^*x*^ complexes which catalyse hydrogenation of biomolecules such as pyruvate and quinones in aqueous media, and generate biologically important species such as H_2_ and H_2_O_2_. Organometallic catalysts can achieve enantioselectivity, and moreover can be active in living cells, which is surprising on account of the variety of poisons present. Such catalysts can induce reductive stress using formate as hydride source or oxidative stress by accepting hydride from NAD(P)H. In some cases, photocatalytic redox reactions can be induced by light absorption at metal or flavin centres. These artificial transformations can interfere in biochemical pathways in unusual ways, and are the basis for the design of metallodrugs with novel mechanisms of action.

## Abstract 2

Recent developments in transfer hydrogenation catalysis and photocatalysis in cancer cells by synthetic metal complexes are reviewed. They offer exciting new ways to modulate biochemical pathways for drug development and biotechnology.

## Graphical abstract

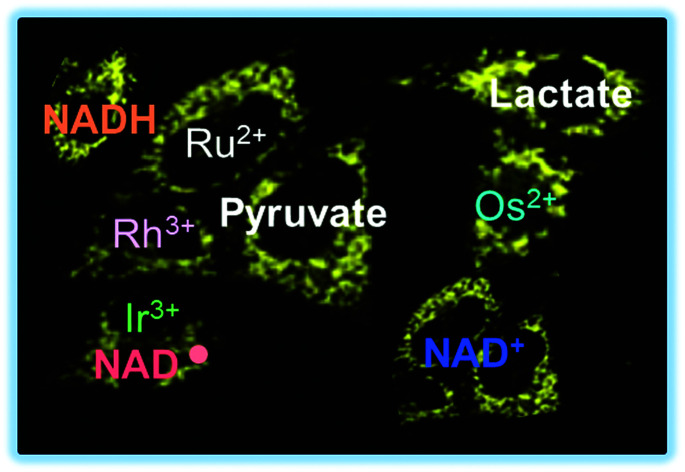

## Introduction

Metal complexes have long since attracted significant attention as catalysts for various chemical transformations.^1–6^ In most of these metal complex-catalyzed reactions, the conditions are well defined, such as concentration of catalyst and substrate, solvent, and temperature. Typically non-polar and non-aqueous solvents are used and reactions are generally carried out in the absence of any competitive reactants or catalysts.^1–6^ However, in spite of all the precautions, catalysts are often poisoned.^7–10^ On the other hand, nature very carefully uses enzymes with specific metal-ion active sites to catalyze a wide range of intracellular bio-chemical reactions essential for life.^11,12^ These metalloenzymes catalyse reactions in living cells in which the conditions and environment are extremely complicated.^7,11,12^ Metal ions such as iron, copper, zinc, and manganese are encapsulated by proteins, with engineered reaction sites which allow substrate recognition, a reactive ‘entatic/ecstatic’ state, and protection of the active metal ion against poisoning.^11–13^

There is interest in the design of artificial metalloenzymes involving a synergistic combination of enzymology and synthetic inorganic chemistry,^14–18^ but these artificial metalloenzymes are not very cost effective and generally require careful handling and storage.^19^ Low-molecular-weight metal complexes are attractive as mimics of metalloenzymes.^7,11,19,20^ However, without a large protein scaffold, small catalysts may not possess substrate specificity and intracellular nucleophiles might readily poison the active catalyst.^7,11,19,20^ However, recently, some progress has been achieved with transfer hydrogenation catalysis in cells by some second- and third-row transition metal low-spin d^6^ Ru^II^, Rh^III^, Ir^III^ and Os^II^ complexes.^21–38^

Here we describe recent work on catalytic transfer hydrogenation in cells. Several enzymes are known to be involved in the reduction of NAD(P)^+^ to NAD(P)H, or oxidation of NAD(P)H to NAD(P)^+^ (Table 1).^39–50^ Intracellular NAD^+^/NADH inter-conversion has been achieved by numerous organometallic Ru^II^, Rh^III^, Ir^III^, Os^II^ complexes *via* a transfer hydrogenation pathway in the presence of a hydride donor,^21–36^ including catalytic reduction of pyruvate to lactate in cells,^37^ and in-cell photo-redox catalysis of NADH oxidation.^51^ We discuss these recent developments including detailed mechanisms of action, along with recent work on in-cell photo-catalytic reduction of metal complexes by flavines.^20,52–55^ Coenzymes NAD^+^/NADH play important roles in maintaining the intracellular redox balance and mitochondrial electron transport chain, and such in-cell transfer hydrogenation catalysts might find application in biotechnology and therapy, for example as catalytic anticancer drugs.

**Table 1:** Some endogenous enzymes that transfer hydride to substrates

Enzyme	Reaction catalyzed	Ref.
Isocitrate dehydrogenase	Isocitrate + NAD^+^ ⇌ 2-oxoglutarate + CO_2_ + NADH + H^+^	39
	Isocitrate + NADP^+^ ⇌ 2-oxoglutarate + CO_2_ + NADPH + H^+^	
Oxoglutarate dehydrogenase complex	α-Ketoglutarate + NAD^+^ + CoA → Succinyl CoA + CO_2_ + NADH	40
Malate dehydrogenase	Malate + NAD^+^ → oxaloacetate + NADH + H^+^	41
Alcohol dehydrogenases	CH_3_CH_2_OH + NAD^+^ → CH_3_CHO + NADH + H^+^	42
NAD-dependent formate dehydrogenases	Formate + NAD^+^ ⇌ CO_2_ + NADH + H^+^	43
NADH dehydrogenase	NADH + H^+^ + acceptor ⇌ NAD^+^ + reduced acceptor	44
NADH:ubiquinone reductase	NADH + H^+^ + ubiquinone ⇌ NAD^+^ + ubiquinol	45
Glucose dehydrogenases	Beta-d-glucose + NAD(P)^+^ ⇌ d-Glucono-1,5-lactone + NAD(P)H + H^+^	46
Non-phosphorylating glyceraldehyde 3-phosphate dehydrogenase	Glyceraldehyde-3-phosphate + NADP^+^ + H_2_O → 3-phosphoglycerate + NADPH + H^+^	47
Ferredoxin–NADP^+^ reductase	Reduced ferredoxin + NADP^+^ + H^+^ ⇌ oxidized ferredoxin + NADPH	48
Adrenodoxin reductase	NADPH + oxidized adrenodoxin → reduced adrenodoxin + NADP^+^ + H^+^	49
NADPH oxidase	NADPH + 2O_2_ ⇌ NADP^+^ + 2O_2_^−^ + H^+^	50

## Enzymes that transfer hydride to substrates

Several enzymes are known to transfer hydride to NAD^+^ or NADP^+^ from a hydride source, Table 1.^39–43,46–48^ For example, isocitrate dehydrogenase transfers hydride to NAD^+^ from isocitrate,^39^ alcohol dehydrogenases transfer hydride from ethanol to NAD^+^.^42^ These bio-catalytic reactions are associated with the liberation of CO_2_ and are integral parts of cellular metabolism and the citric acid cycle.^56^ A few enzymes catalyse oxidation of NADH *via* a transfer hydrogenation, such as NADH:ubiquinone reductase which oxidizes NADH to NAD^+^ and transfers hydride to ubiquinone to generate ubiquinol.^45^ Interestingly, NADPH oxidase transfers hydride from NADPH to molecular oxygen.^50^ All these enzymes are important for maintaining the intracellular redox and proton balance.

## Hydride sources in cells

**Fig. 1:**
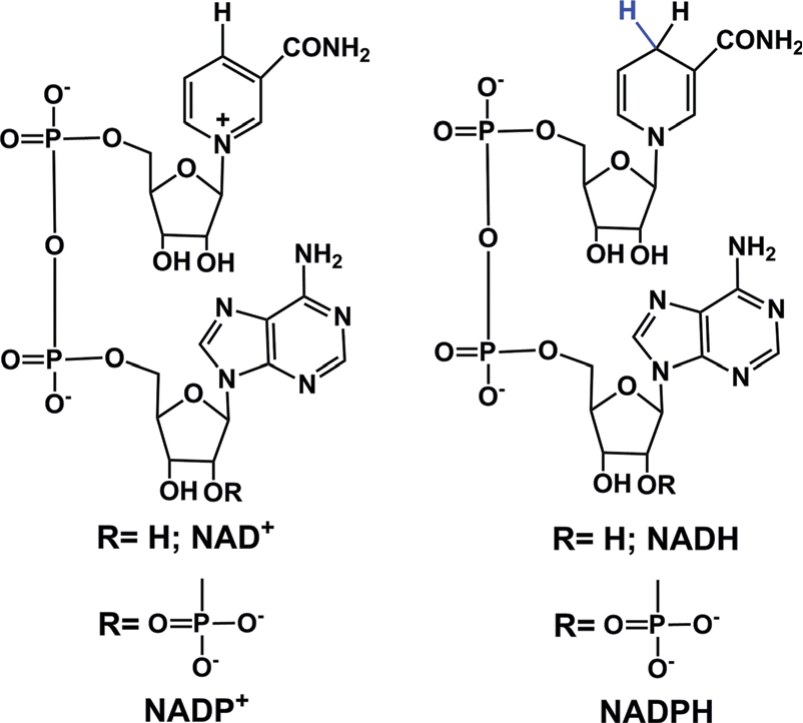
Structures of NAD^+^/NADH and NADP^+^/NADPH.

**Fig. 2:**
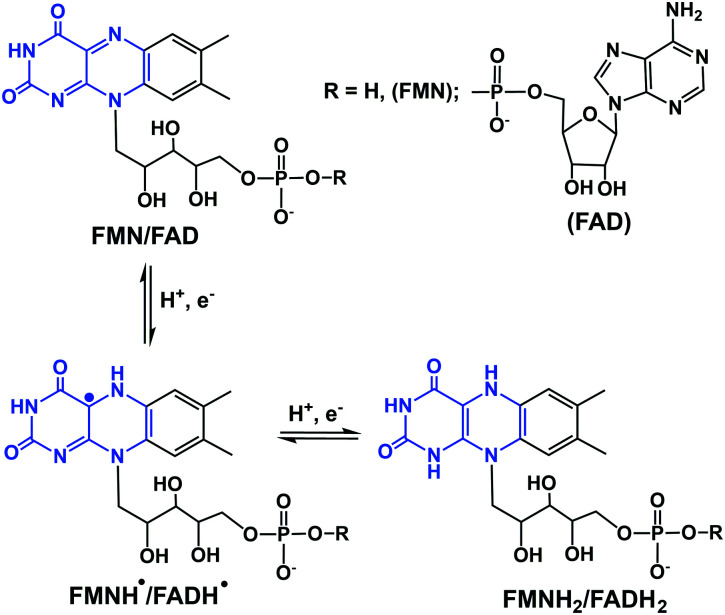
One- and two-electron redox reactions of flavins. The key region involved in electron/hydride transfer is highlighted in blue.

In eukaryotic cells, the main direct sources of endogenous hydride are reduced nicotinamide adenine dinucleotide (NADH, Fig. 1), reduced nicotinamide adenine dinucleotide phosphate (NADPH, Fig. 1) and reduced flavin adenine dinucleotide (FADH_2_, hydroquinone form, Fig. 2).^30,44,45,49,50,57–61^ Their electrochemical potentials, intracellular concentrations and distributions are summarized in Table 2.

**Table 2:** Electrochemical potential, intracellular concentration and distribution of the common hydride sources in cells

Hydride	Potential	Concentration	Distribution
NADH	*E*_NAD^+^/NADH_ = −0.32 V	100 to 200 μM	Cytosolic component: NADH shuttled from cytosol to mitochondria by malate-aspartate shuttle or glycerol 3-phosphate shuttle
NADPH	*E*_NADP^+^/NADPH_ = −0.32 V	No definite range	Cytosol and mitochondria
FADH_2_	*E*_FAD/FADH_2__ = −0.22 V	No definite range	Cytosol and mitochondria

In cells, NADH is often generated by the reduction of its oxidized form, NAD^+^ during metabolism and in the citric acid cycle by the enzymes isocitrate dehydrogenase, oxoglutarate dehydrogenase complex and malate dehydrogenase.^39–41^ Other enzymes such as alcohol dehydrogenases, NAD-dependent formate dehydrogenases (in methylotrophic yeast and bacteria) are also known to reduce NAD^+^ to NADH.^42,43^ NADH is an important hydride donor in cells and donates hydride to flavin mononucleotide (FMN) in complex I of the mitochondrial electron transport chain.^57–60^ NADH first binds to the hydrophilic domain of complex I which contains FMN, and then transfers two electrons to FMN, the prosthetic group of the NADH reductase,^62,63^ which is reduced to FMNH_2_. The electron acceptor is the isoalloxazine ring of FMN.^64,65^ NADH is oxidized to NAD^+^ by the enzymes NADH dehydrogenase and NADH:ubiquinone reductase, or during oxidative phosphorylation to generate ATP.^44,45^

Phosphorylation of NAD^+^ by NAD^+^ kinases leads to the synthesis of NADP^+^, which in turn is reduced to NADPH by glucose-6-phosphate dehydrogenase (G6PDH) in the first step of the pentose phosphate pathway.^66^ The conversion of NADP^+^ to NADPH is also carried out by enzymes such as isocitrate dehydrogenase (in the citric acid cycle), glucose dehydrogenases, non-phosphorylating glyceraldehyde 3-phosphate dehydrogenase (in plants, algae, and bacteria), transhydrogenases and ferredoxin–NADP^+^ reductase (in plants; involved in photosynthesis).^39,46–48,67^ NADPH is also an important hydride donor, for several biosynthetic reactions and the regeneration of glutathione.^67^ The enzyme adrenodoxin reductase, present in most common organisms, oxidizes NADPH to NADP^+^.^49^ In the plasma membrane and in the membranes of phagosomes, NADPH oxidase can oxidize NADPH.^68,69^

The other important endogenous hydride donors are FMNH_2_ and FADH_2_.^61,70,71^ whereas NADH and NADPH are involved only in two-electron (hydride) transfer, the flavins (FMNH_2_ and FADH_2_) can mediate both the one and two electron transfer processes as shown in the Fig. 2. Generally, during an enzymatic process, the oxidized (FMN/FAD), semiquinone (FMNH˙/FADH˙) and reduced (FMNH_2_/FADH_2_) forms undergo reversible interconversion (Fig. 2).^71–76^ FMNH_2_ donates hydride to the coenzyme Q10 (ubiquinone) and is oxidized back to FMN.^76^ whereas FADH_2_ donates hydride to complex II of the mitochondrial electron transport chain.^77^

The electron/H^+^ and hydride transfer processes for NAD(P)H and FMNH_2_ and enzymes are complex processes. For example, high-resolution X-ray crystallographic hydride transfer studies in the ferredoxin:NADP^+^ reductase (FNR) family reported by Kean *et al.* show the mobility of nicotinamide's C4 atom in the FNR:NADP^+^ complex, which results in the boat-like conformation of the nicotinamide ring. This conformational change in turn enhances hydride transfer.^48^

**Fig. 3:**
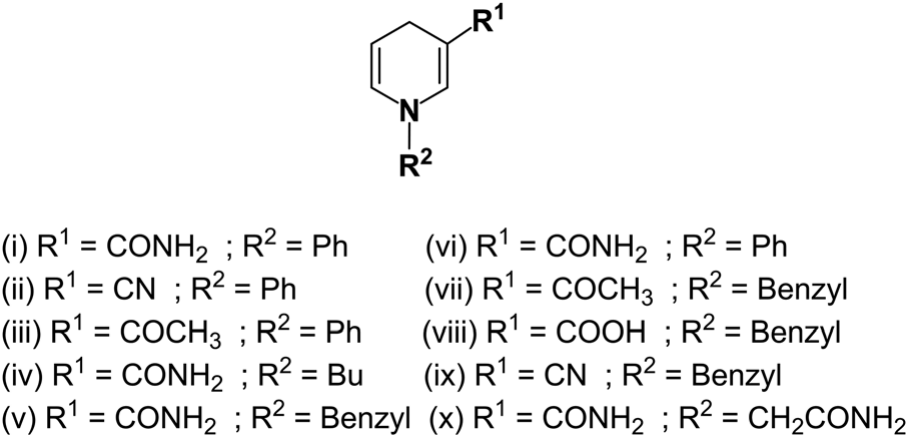
Structures of synthetic NADH mimics.^78,80–82^

Small molecules as functional mimics of NADH have attracted significant attention for recharging cofactor-dependent enzymes, and understanding the pathways of naturally occurring biochemical reactions.^78–83^ For halogenation activity, tryptophan 7-halogenase needs FADH_2_ which is generated from the reaction of FAD with NADH by a flavin reductase.^78^ van Pée *et al.* catalytically regenerated FADH_2_ from FAD using the small organometallic ion [Cp*Rh(bpy)(H_2_O)]^2+^ as catalyst and formate as the electron donor.^79^ Sewald *et al.* employed the NADH mimics shown in Fig. 3 (compounds i–iv) to achieve chlorination of l-tryptophan using FAD-dependent halogenase.^78^ These NADH mimics take care of FADH_2_ regeneration from FAD.^78^ Scrutton and coworkers reported the excellent efficiency of the NAD(P)H mimics (iv–viii, Fig. 3) in Ene reductases catalyzed reactions.^80^ Compounds iv, v, vii–ix (Fig. 3) as synthetic NADH mimics for enhanced enoate reductase catalyzed reactions are reported by Hollmann *et al.*^81^ Interestingly, these mimics do not decrease the enzymatic activity or stereo-selectivity of the C=C bio-reductions. Knox *et al.* used 1-carbamoylmethyl-3-carbamoyl-1,4-dihydropyridine (compound x in Fig. 3) as NADH mimic to activate the NAD(P)H quinone oxidoreductase 2 which finally activates cancer prodrug 5-(aziridin-1-yl)-2,4-dinitrobenzamide.^82^ These examples illustrate the potential for synthetic NADH mimic in bio-catalysis and biomedical applications.

## Transfer hydrogenation catalysis

Transfer hydrogenation catalysis by metal complexes involves transfer of hydride from a donor to an acceptor substrate *via* a metal-hydride intermediate.^84–86^ This reaction is well known in synthetic organic chemistry for reduction of C=C, ketones (by Noyori's ruthenium arene complexes), and imines in non-aqueous media in the presence of hydride donors such as formate or isopropanol.^86^

Several Ru^II^, Rh^III^ and Ir^III^ complexes can achieve regioselective reduction of NAD^+^ to 1,4-NADH in aqueous solution with sodium formate as the hydride source.^87–93^ In 1988, Steckhan *et al.* reported the regioselective reduction of NAD^+^ in aqueous media by [Rh(Cp*)(2,2′-bipyridine)(H_2_O)_2_]^+^*via* transfer of hydride from formate.^87^ They also elucidated the mechanism of NAD^+^ reduction by bipyridine-chelated Cp*Rh^III^ complexes.^87^ Catalytic reduction of NAD^+^ by a series of phenanthroline-chelated Ru^II^, Rh^III^ and Ir^III^ catalysts in aqueous media has been reported by Süss-Fink *et al.*^90^ Diamine Ru(ii)–arene complexes also catalyse NAD^+^ reduction in aqueous solution and isotope studies indicate that the formation of the Ru–H hyudride species is the rate-limiting step in the catalytic cycle.^92,93^

The establishment of transfer hydrogenation catalysis in aqueous solution under mild conditions provides a basis for extending the scope of catalysis to cells for the reduction of biomolecules such as NAD^+^ and pyruvate. This is challenging on account of the presence of numerous nucleophilic biomolecules and as well as oxidants and reductants, which might potentially poison the active catalyst.

## In-cell catalytic reduction of NAD^+^

**Fig. 4:**
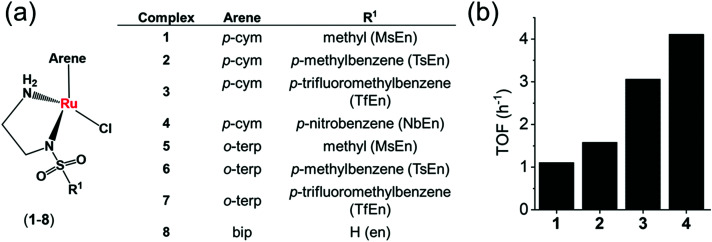
(a) Structures of sulfonamidoethylenediamine Ru^II^ transfer hydrogenation catalysts **1–8**.^21^ (b) Turnover frequencies (TOF) for NAD^+^ reduction in MeOH-d_4_/D_2_O (5 : 1 v/v) by complexes **1–4** determined by ^1^H NMR ([Ru complex]: 0.44 mM; [NAD^+^]: 0.88 mM; [formate]: 11.02 mM; molar ratio 1 : 2 : 25).^21^

Coenzymes NAD^+^ and NADH control >400 enzymatic redox reactions which involve their inter-conversion.^94–96^ In cells, the conversion of NAD^+^ to NADH usually involves transfer hydride from a substrate to NAD^+^.^38–43^ In 2015, such reduction was achieved in living cells using arene Ru^II^ Noyori-type transfer hydrogenation catalysts (Fig. 4a) containing a chelated sulfonamidoethylenediamine ligand co-incubated with the hydride donor, formate to reduce NAD^+^ to NADH.^21^ In MeOH-d_4_/D_2_O (2 : 9 v/v) or in D_2_O, complexes **1–4**, catalytically and regioselectively reduce NAD^+^ to NADH, as was evident from the ^1^H NMR analysis. Higher catalytic activity (higher turnover frequency, TOFs) was observed with the more electron-withdrawing sulfonamides (NbEn (**4**) > TfEn (**3**) > TsEn (**2**) > MsEn (**1**)) (Fig. 4b). Complexes **5–7** gave rise to extremely fast NAD^+^ reduction and the reactions were completed before the first ^1^H NMR spectrum could be recorded indicating that the *o*-terphenyl (*o*-terp) complexes were more catalytically active than the *p*-cym complexes. Aquation of the complexes (replacement of Cl by H_2_O, Fig. 4a) was very fast. The Ru–H intermediate was detectable by ^1^H NMR with a peak at −5.5 ppm and existence of the formate adduct was confirmed by the mass spectral analysis. Ru–H formation occurs in three steps (i) the initial Ru–Cl bond hydrolyses, (ii) formate binds to Ru^II^*via* a carboxylate oxygen, and (iii) formate re-orientates to facilitate transfer of hydride to Ru(ii) with the release of CO_2_.

**Fig. 5:**
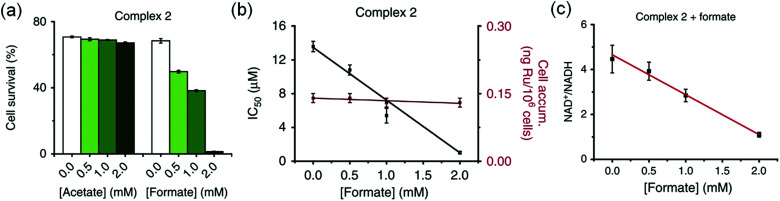
(a) Percentage survival of A2780 human ovarian cancer cells co-incubated with complex **2** and various concentrations of sodium acetate or sodium formate. (b) IC_50_ values of complex **2** when co-incubated with sodium formate and the intracellular uptake of Ru under similar conditions. (c) Linear correlation of sodium formate concentration and the NAD^+^/NADH ratio when co-administered with complex **2**. Figure reproduced from ref. 21; J. J. Soldevila-Barreda *et al.*, *Nat. Commun*., 2015, **6**, 6582, published by Springer Nature.

The complexes showed anticancer activity against A2780 human ovarian cancer cells with the half maximal inhibitory concentration (IC_50_) values in the range of 2.2–21.2 μM. When co-administrated with formate, the anticancer profile of complexes **1–7** improved (Fig. 5a). Such an effect was not observed with acetate, which is not a hydride donor (Fig. 5a). The extent of lowering of IC_50_ values (increase in cytotoxicity) was directly proportional to the formate concentration (Fig. 5b), suggesting a direct contribution of catalytic transfer hydrogenation to the anticancer profile. Such a direct contribution was confirmed for complex **2** which significantly decreased the intracellular NAD^+^/NADH ratio when cells were co-incubated with non-toxic doses of formate (Fig. 5c). Moreover, formation of coenzyme NADH induced reductive stress, a new and unusual mechanism of action for an anticancer agent, was observed. Thus this mechanism might be effective for overcoming cisplatin resistance, a major clinical problem.

**Fig. 6:**
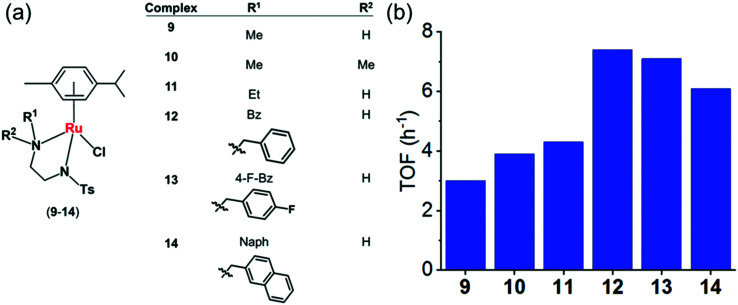
(a) Structures of sulfonamidoethylenediamine Ru^II^ transfer hydrogenation catalysts **9–14**.^22^ (b) Turnover frequencies (TOF) for reduction of NAD^+^ by formate catalyzed by complexes **9–**1**4**, determined by UV-vis spectroscopy (84 μM complex in MeOH/H_2_O 1 : 9 v/v, 102 mM sodium formate and 510 μM NAD^+^ in H_2_O).^22^

To establish structure–activity relationships, we studied six pseudo-octahedral neutral Ru^II^ sulfonamidoethylenediamine complexes of the type [(η^6^-*p*-cym)Ru(*N*,*N*′)Cl] (**9–14**) (Fig. 6a).^22^

These complexes catalyse the reduction of NAD^+^ to 1,4-NADH regioselectively when formate is used as the hydride donor. The catalytic activity is highly dependent on the electronic and steric effects of the *N*-substituent, the bulkier the substituent, the higher the rate of reduction (Fig. 6b). The rate of NAD^+^ reduction is dependent on pH* (deuterated solvent) and was highest between pH* 6–7.5. An increase in formate concentration increased the rate of reduction.

**Fig. 7:**
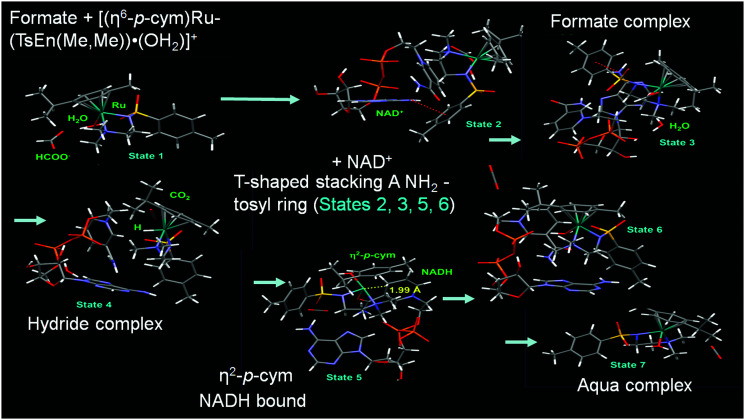
Catalytic cycle for NAD^+^ reduction by the complexes **9–14** based on DFT modelling. Reproduced from ref. 22; F. Chen *et al.*, *Dalton. Trans*., 2018, **47**, 7178, published by The Royal Society of Chemistry.

DFT studies indicated a mechanism involving the initial replacement of an aqua ligand by formate, followed by H^−^ transfer to Ru^II^ and finally to NAD^+^. Furthermore, specific interactions between the NAD^+^ and the aqua complex were evident from the modelling and probably allow a pre-organisation *via* interaction of the aqua ligand, formate and the pyridine ring of NAD^+^ (Fig. 7). The complexes showed antiproliferative activity towards human ovarian cancer cells, which was further increased by 20–36% on co-administration with 2 mM sodium formate.

**Fig. 8:**
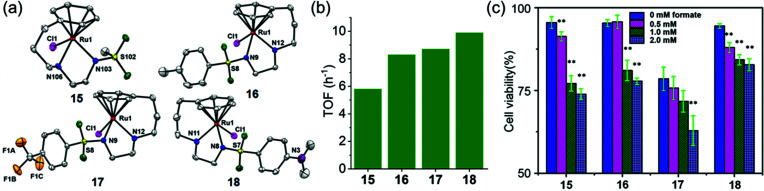
(a) ORTEP diagrams of the X-ray crystal structures of tethered Ru^II^ catalysts **15–18** with sulfonyl substituents on the diamine. Figure reproduced from ref. 23; F. Chen *et al.*, *Organometallics* 2018, **37**, 1555, published by The American Chemical Society. (b) TOFs for complexes **15–18** for NAD^+^ reduction in MeOH-d_4_/D_2_O (1 : 9 v/v) determined by UV-vis spectroscopy (final concentrations: Ru complex 28 μM, NAD^+^ 170 μM, NaHCO_2_ 34 mM, molar ratio 1/6/1200)).^23^ (c) Cell viability of A2780 cancer cells when incubated with complexes **15–18** (at equipotent 1/3 × IC_50_ concentrations) and various sodium formate concentrations (0, 0.5, 1.0, and 2.0 mM) for 24 h. The figure is reproduced from ref. 23; F. Chen *et al.*, *Organometallics* 2018, **37**, 1555, published by The American Chemical Society.

Half-sandwich and tethered Ru^II^ complexes where the diamine ligand and the η^6^–arene ring are directly connected, provide control over the spatial positions of ethylenediamine substituents which results in extra stability of the complexes,^97,98^ are also transfer hydrogenation catalysts for reduction of ketones and imines.^99,100^ The neutral tethered Ru^II^ complexes [Ru(η^6^-Ph(CH_2_)_3_-ethylenediamine-*N*-R)Cl] where R is methanesulfonyl (Ms, **15**), toluenesulfonyl (Ts, **16**), 4-trifluoromethylbenzenesulfonyl (Tf, **17**), and 4-nitrobenzenesulfonyl (Nb, **18**), (Fig. 8a)^23^ are potent transfer hydrogenation catalysts for the reduction of NAD^+^ to NADH with formate as hydride donor both in aqueous solution (TOFs/h = 3.8–10) and in cancer cells. In aqueous media, the reduction can be monitored by following the absorbance of NADH at 340 nm using UV-visible spectroscopy.^101^ Substituents on the ethylenediamine ligand control the catalytic activity, and the turnover frequency decreases in the order Nb (**18**) > Tf (**17**) > Ts (**16**) > Ms (**15**) (Fig. 8b) again showing that more strongly electron-withdrawing groups enhance hydride transfer.

These complexes are moderately cytotoxic to human lung (A549), ovarian (A2780), breast (MCF7) and hepatocellular (HEPG2) cancer cells. Interestingly, up to 22% enhancement of cytotoxicity of the complexes was observed on co-incubation with non-toxic doses of sodium formate (0.5–2 mM) (Fig. 8c) indicating that reduction of intracellular NAD^+^ may contribute significantly to the anticancer activity.

**Fig. 9:**
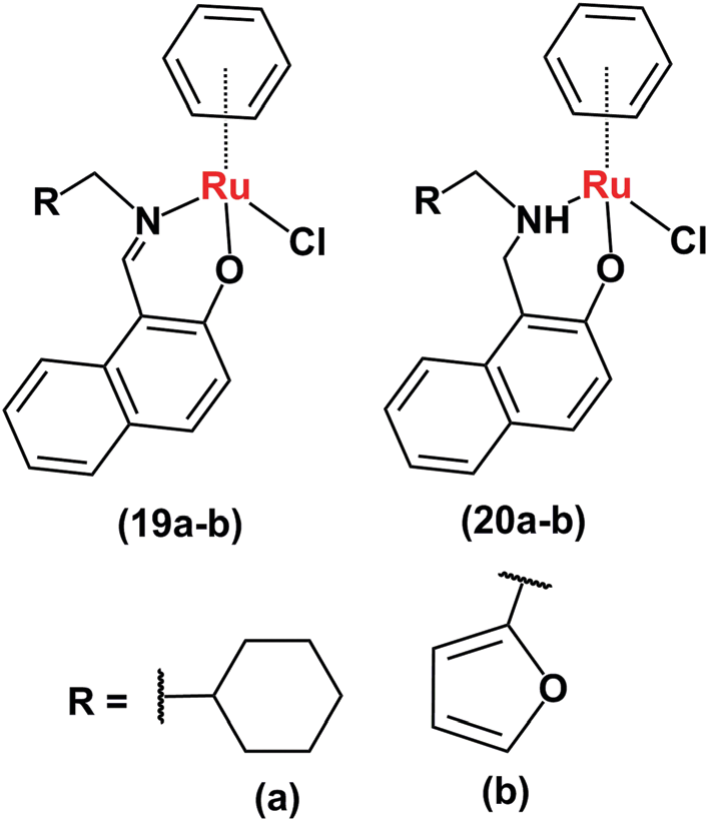
Ruthenium arene complexes **19** and **20**.^24^

Ruthenium(ii)–arene complexes with bidentate Schiff base ligands (**19a**, **19b**Fig. 9) or their reduced analogues (**20a** and **20b**) also have anticancer activity and an ability to reduce NAD^+^.^24^ In comparison to the Schiff base complexes, the corresponding amine complexes exhibit improved anticancer activity against various human cancer cell lines, as well as higher rates of catalytic NAD^+^ reduction. This study shows that simple ligand modifications (reduction of an imine) can significantly alter both the biological and catalytic activities.

**Fig. 10:**
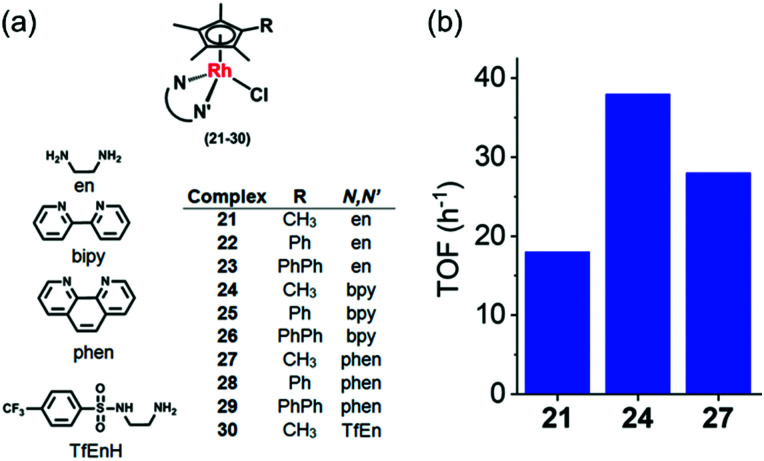
(a) Rh(iii) catalysts **21–30** for the reduction of NAD^+^ in the presence of sodium formate as hydride source.^25^ (b) TOFs for reduction of NAD^+^ by formate, catalyzed by Cp* Rh^III^ complexes **21**, **24** and **27** in MeOH-d_4_/D_2_O (1 : 4 v/v) determined by ^1^H NMR (6–9 mol equiv. NAD^+^ and 25 mol equiv. formate).^25^

Rh^III^ complexes can catalyse transfer hydrogenation for NAD^+^ reduction to NADH using formate as the hydride source, under biologically-relevant conditions, for example [(Cp^*x*^)Rh(*N*,*N*′)(Cl)]^+^ (**21–30**, Fig. 10a), where *N*,*N* = ethylenediamine (en), 2,2′-bipyridine (bpy), 1,10-phenanthroline (phen) or *N*-(2-aminoethyl)-4 (trifluoromethyl)benzenesulfonamide (TfEn), and Cp^*x*^ = Cp*, 1-phenyl-2,3,4,5 tetramethylcyclopentadienyl (Cp^*x*Ph^) or 1-biphenyl-2,3,4,5-tetramethyl cyclopentadienyl (Cp^*x*PhPh^).^25^

The structure activity relationship showed that the *N*,*N*-chelated ligand can control the catalytic activity which decreased in the order of bpy (**24**) > phen (**27**) > en (**21**) as shown in the Fig. 10b. [Cp*Rh(bpy)Cl]^+^ (**24**) was the most efficient catalyst with a TOF of 37.4 ± 2 h^−1^ in aqueous solution. Interestingly, complexes **21–29** were able to catalytically reduce pyruvate to lactate using formate as the hydride donor. Preference for the reduction of NAD^+^ over pyruvate was also observed. Remarkably, when co-incubated with non-toxic doses of formate, the anticancer activity of complex **23** in A2780 cancer cells increased by *ca.* 50%, indicating that transfer hydrogenation may induce reductive stress in cancer cells.

## Catalytic oxidation of intracellular NADH

The catalytic reduction of intracellular NAD^+^ to NADH in the presence of a hydride donor can lead to the alteration of intracellular redox homeostasis and cell death. The reverse process, oxidation with NADH as the hydride donor is also achievable both in aqueous solution and in cells.^102–105^ For example in aqueous solution, [*C*,*N*]-cyclometallated Ir(iii) complexes can convert NADH to NAD^+^ under weakly basic conditions.^102^ Also [Ir^III^(Cp*)(4-(1*H*-pyrazol-1-yl-κ*N*^2^)benzoic acid-κ*C*^3^)(H_2_O)]_2_SO_4_ catalyses the reduction of 2,3-dimethoxy-5-methyl-1,4-benzoquinone (Q_0_), an ubiquinone coenzyme analogue, by NADH to the corresponding reduced form (Q_0_H_2_) in aqueous solution at room temperature.^103^ Also [Cp*Ir(pica)Cl] where pica is picolinamidate = κ^2^-pyridine-2-carboxamide, catalyses the dehydrogenation of β-NADH in slightly acidic aqueous solution,^104^ not only to the expected β-NAD^+^ but also α-NAD^+^, nicotinamide and 1,2,5,6-tetrahydronicotinamide.^104^

**Fig. 11:**
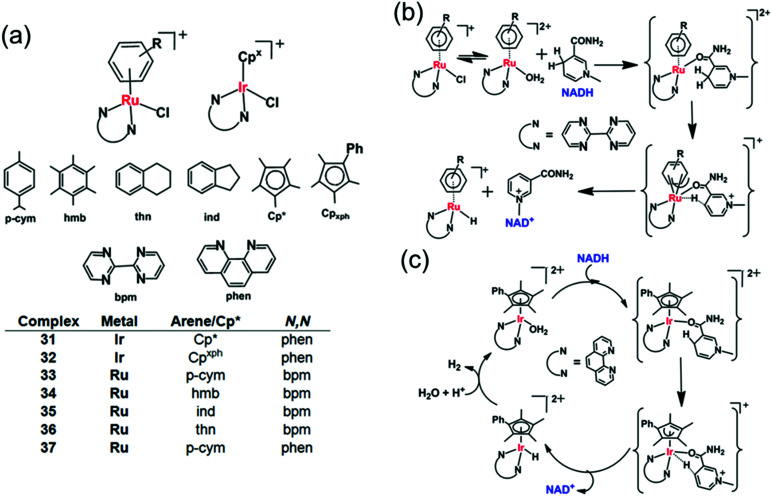
Cyclopentadienyl Ir^III^ (**31**, **32**) and arene Ru^II^ transfer hydrogenation catalysts (**33–37**) for NADH oxidation.^26^ Proposed mechanism for transfer of hydride from 1,4-NADH to (b) Ru^II^ and (c) Ir^III^ complexes involving nicotinamide carbonyl coordination and arene/Cp ring slippage.^26^

Half-sandwich cyclopentadienyl iridium(iii) and arene ruthenium(ii) complexes [(Cp^*x*^)Ir(phen)(H_2_O)]^2+^ (**31**, **32**) and [(arene)Ru(*N*,*N*′)Cl]^+^ (**33–37**) (Fig. 11a) can reduce ketones using NADH as the hydride source.^26^ Moreover, under physiological conditions, these complexes reduce pyruvate to lactate, a reduction carried out by the enzyme lactate dehydrogenase *in vivo* using NADH as a cofactor.^106^ The Ir(iii) complexes [(Cp*/Cp^*x*Ph^)Ir(phen)(H_2_O)]^2+^ (**31**, **32**) behave as robust catalysts for H_2_ generation from NADH. Under physiologically relevant conditions (pH 7.4, 310 K), a TON of 75 after 24 h and a TOF up to 4.3 h^−1^ were achieved. Complex **32** almost doubled the NAD^+^/NADH ratio level in A2780 cancer cell lysates indicating that the complex can modulate the intracellular redox balance, and that catalytic oxidation of NADH can be achieved in cells by transfer hydrogenation catalysts.

DFT modelling suggests that the mechanism for the oxidation of NADH to NAD^+^ involves transfer of hydride from NADH to the Ru^II^/Ir^III^ centre *via* formation of a six-membered transition state, by a coordination site which becomes vacant by a ring-slippage mechanism (Fig. 11b and c).^107,108^ Notably the formation of both Ru–H and Ir–H was detected by ^1^H NMR, at −7.44 ppm and −11.3 ppm, respectively.

**Fig. 12:**
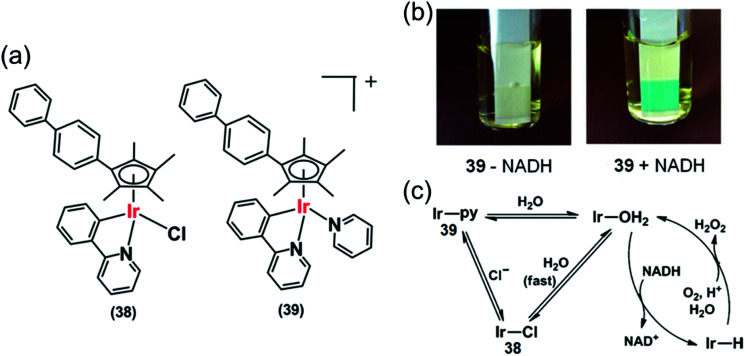
(a) Ir^III^ phenylpyridine transfer hydrogenation catalysts **38** and **39**.^27^ (b) Detection of H_2_O_2_ in a solution of **39** + NADH in MeOH/H_2_O (3 : 7, v/v) at 310 K by Quantofix peroxide test sticks. (c) Proposed catalytic circle for the production of H_2_O_2_ by **38** and **39**. Reproduced from ref. 27 with permission.

Organoiridium(iii) complexes [(Cp^*x*biph^)Ir(phpy)(Cl)] (**38**) and [(Cp^*x*biph^)Ir(phpy)(py)]^+^ (**39**) (Fig. 12a) where Cp^*x*biph^ = biphenyltetramethylcyclopentadienyl, phpy = phenylpyridine and py = pyridine, can transfer the hydride from NADH *via* iridium-hydride intermediates to O_2_, a possible route for intracellular H_2_O_2_ generation.^27^ The activity of these complexes is analogous to that of NADPH oxidase which transfers hydride from NADPH to O_2_ in cells.^50^ These complexes were active against a wide range of cancer cell lines in the National Cancer Institute NCI-60 human cancer cell screen.

Hydride transfer from NADH to Ir(iii) was much slower for pyridine complex **39** than for chlorido complex **38**, attributable to slower hydrolysis rate of **39**. Importantly, H_2_O_2_ generation was also detected from the appearance of a blue colour on a H_2_O_2_ test stick in the presence of NADH (Fig. 12b) indicating transfer of hydride from NADH to O_2_*via* an Ir–H intermediate (Fig. 12c) detectable by ^1^H NMR.

**Fig. 13:**
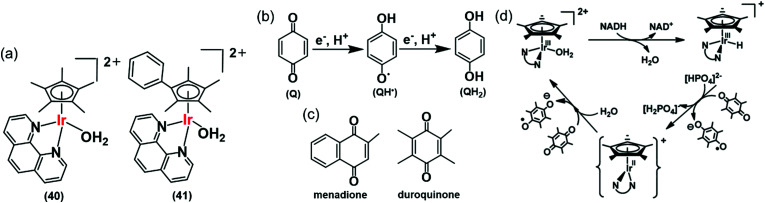
(a) Ir^III^ TH catalysts **40** and **41** for the reduction of quinones. (b) One and two electron reduction of quinones leading to semiquinone and hydroquinone, respectively. (c) Structures of menadione and duroquinone.^28^ (d) Catalytic cycle for the reduction of quinones by the NADH/Ir^III^ system. Reproduced form ref. 28 with permission.

Hydride can also be transferred from NADH to quinones by the Ir(iii) complexes [(Cp*)Ir(phen)(H_2_O)]^2+^ (**40**), and [(Cp^*x*ph^)Ir(phen)(H_2_O)]^2+^ (**41**) (Fig. 13a).^28^ Quinones (Q) have important roles carrying electrons in the mitochondrial electron-transport chain.^109,110^ Quinones undergo one- or two-electron reduction to the corresponding semiquinones (QH˙) or hydroquinones (QH_2_), respectively (Fig. 13b). In cells, quinones are generally reduced by coenzyme NAD(P)H in the presence of either NADH ubiquinone oxidoreductase or NADPH cytochrome P-450 reductase, or NADH cytochrome *b*_5_.^73,111–113^ Complex **41** reduces duroquinone and menadione (vitamin K_3_) (Fig. 13c) with a TON of 56.6 and TOF of 12.4 h^−1^ (in phosphate buffer at pH 7.2) to the corresponding semiquinone radicals, which were detected by EPR.

The reduction of quinones by NADH occurs *via* generation of Ir–H intermediates. DFT calculations suggested two one-electron transfers, the first to form the semiquinone and a transient Ir^II^ state, which in turn transfers a second electron to a second quinone, forming the second quinone radical with the regeneration of active Ir^III^ species (Fig. 13d). Ir^II^ is a relatively rare oxidation state, but has been reported in the literature.^114–116^ As a consequence, such organometallic complexes have the potential to convert the two-electron reducing power of NADH into two subsequent one-electron steps. This opens up new ways of diverting biochemical pathways in cells.

**Fig. 14:**
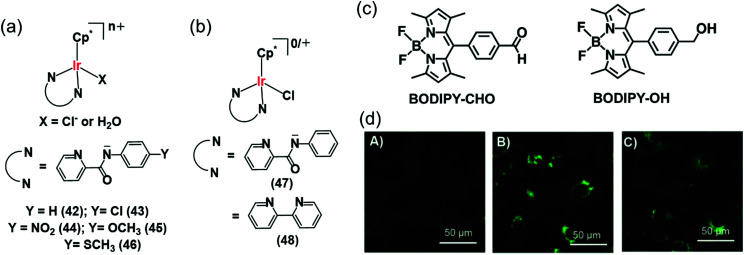
(a) Ir^III^ TH catalysts **42–46** which reduce aldehydes in PBS and cell culture media using NADH as hydride source.^29^ (b) Organometallic Ir^III^ catalysts (**47**, **48**) for intracellular conversion of aldehydes to alcohols.^30^ (c) The fluorogenic BODIPY substrate (BODIPY-CHO) and its reduced form (BODIPY-OH). (d) Confocal microscope images of NIH-3T3 cells treated with (A) BODIPY-CHO (30 μM); (B) BODIPY-OH (30 μM) and (C) BODIPY-CHO (30 μM) + **47** (20 μM). Reproduced from ref. 30 with permission.

Interestingly Do *et al.*, transferred hydride from NADH to aldehydes in PBS or cell culture media (RPMI-1640 or M199) of pH 7.4 at 37 °C, even in the presence of various biomolecules (*e.g.* nucleobases, amino acids, small peptides, carbohydrates) and metal ions (bio-relevant transition metals ions and alkali/alkaline-earth metal ions) using Cp* Ir(iii) complexes of pyridinecarboxamidates (**42–46**) (Fig. 14a).^29^ These complexes were active in hydrogenation of cytotoxic aldehydes responsible for various diseases.

The intracellular conversion of aldehydes to alcohols in living cells can be achieved using Ir(iii) transfer hydrogenation catalysts **47** and **48** and endogenous NADH as the hydride donor (Fig. 14b).^30^ The reduction can be monitored in real time using a fluorogenic BODIPY-CHO substrate (Fig. 14c) and confocal microscopy (Fig. 14d). BODIPY-CHO is not highly fluorescent, but when reduced to BODIPY-OH by intracellular transfer hydrogenation, it becomes strongly fluorescent (Fig. 14c). Such biocompatible reductive chemistry may provide new biotechnological approaches and novel intracellular bio-conjugation strategies.^117^

**Fig. 15:**
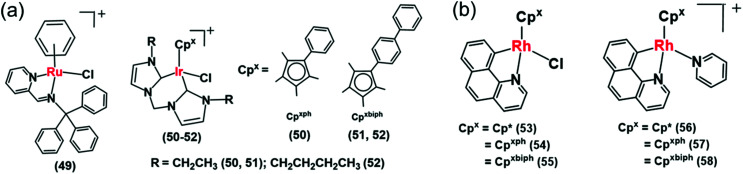
(a) The Ir^III^ iminopyridine and *cis*-carbene transfer hydrogenation catalysts (**49–52**) for NADH oxidation.^31,33^ (b) Cyclometalated Rh^III^ benzo[*h*]quinoline complexes (**53–58**).^36^

Recently Liu *et al.* reported a mitochondria-targeting Ru(ii) complex **49** (Fig. 15a) which showed cytotoxicity against A549 cells *via* NADH oxidation and activation of mitochondrial membrane potential depolarization.^31^ Complex **49** induced overproduction of intracellular ROS possibly by transferring hydride from NADH to O_2_. This complex induced cell apoptosis and arrested the cell cycle at the G_0_/G_1_ phase by cyclin-dependent kinase 4/cyclin D1 inactivation.^31^ They also reported a new class of Ir^III^ complexes (**50–52**, Fig. 15a) of N-heterocyclic carbenes (NHCs) with transfer hydrogenation ability.^33^ Interestingly, the TON of the NHC complex **50** was 2× times higher than the previously discussed C^N analogue, complex **38**.^27^ This may be due to the strong electron-donating ability of the carbenes in complex **50** compared to the C^N ligand in **38**, which can effectively labilize the Ir–Cl bond towards hydrolysis, the activation step of the catalysis.

The introduction of *N*,*C*-chelated ligands can increase the potency of Rh(iii) transfer hydrogenation catalysts for NADH oxidation as in [Cp^*X*^Rh(C^N)Z]^0/+^ (**53–58**), where Cp^*X*^ = Cp*, Cp^ph^, or Cp^biph^, C^N = benzo[*h*]quinoline, and Z = chloride or pyridine (Fig. 15b).^36^ Complex **55** was the most efficient catalyst (TON = 58 and TOF/h = 7.6 in 1.6% MeOH/98.4% phosphate buffer (5 mM, pH 7.4) over 24 h at 310 K) for NADH oxidation and moreover increased the ROS level in A549 lung cancer cells and resulting cytotoxicity. Interestingly, the chlorido complexes **53–55** showed *ca.* 2–4 times higher catalytic activity than their respective pyridine analogues **56–58**. Such a difference in reactivity is due to the much slower hydrolysis of pyridine complexes compared to chloride analogues; hydrolysis is believed to be the activation step of the catalytic process.

## Organo-osmium catalysts

**Fig. 16:**
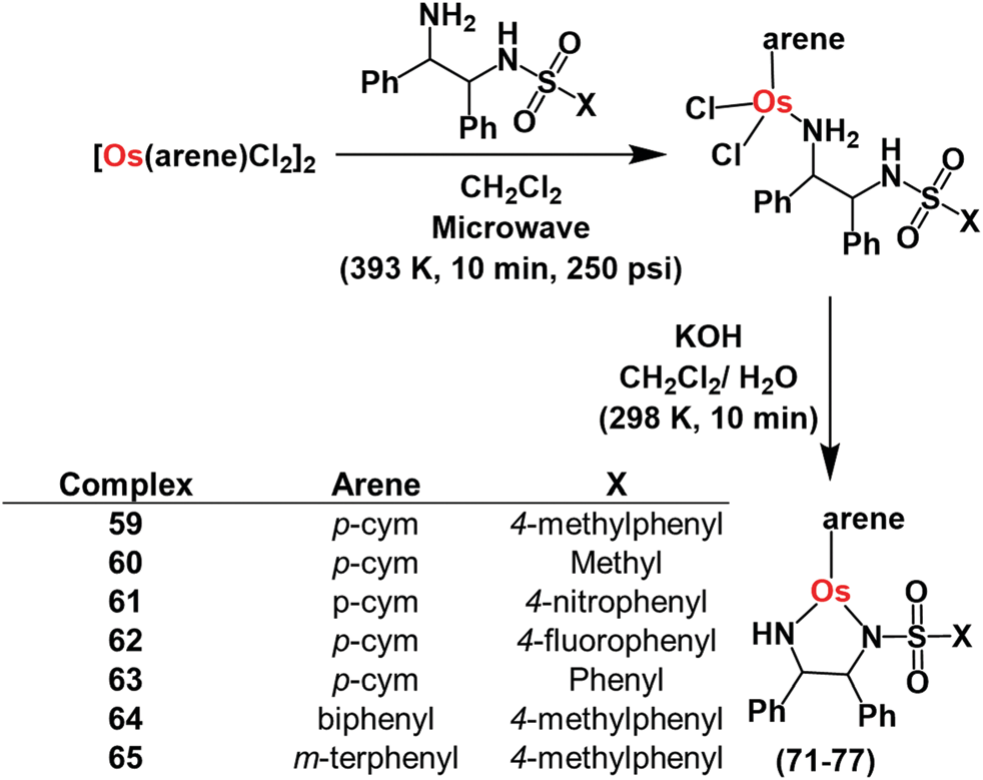
Synthetic route for Os^II^ arene sulfonamidoethylenediamine catalysts **59–65** for pyruvate reduction.

Pyruvate is an important intermediate in metabolic pathways in cells.^118^ In a 3-step process, pyruvate is converted to acetyl-coenzyme A, which generates energy in the Krebs cycle.^118,119^ Hence disturbance of pyruvate metabolism would be expected to generate metabolic disorder. Chiral half-sandwich arene Os(ii) sulfonamidoethylenediamine complexes of the type [Os(arene)(TsDPEN)] where TsDPEN is *N*-(*p*-toluenesulfonyl)-1,2-diphenylethylenediamine (Fig. 16) can catalyse enantioselective reduction of pyruvate.^37^ These 16-electron catalysts have been synthesized as enantiomerically pure compounds by a microwave method as shown in Fig. 16.^37,38^

These 16e catalysts are stable as solids or in solution (including aqueous media), unlike the Ru(ii) analogues which have to be generated from 18e pre-catalysts before use. The Os(ii) complexes reduce acetophenone 3.5 × faster than the Noyori Ru catalyst^37^ in formic acid/triethylamine (5 : 2) azeotrope. They also reduce pyruvate to lactate in the presence of sodium formate as the hydride source *via* transfer hydrogenation in which Os–H is the key intermediate. The turnover frequency of catalysis is highly dependent on formate concentration compared to pyruvate concentration. Interesting, the asymmetric activity of the catalysts is retained with higher enantiomeric excess (83%).

The complexes show moderate to good antiproliferative activitiy against A2780 lung cancer cells (IC_50_= 4–30 μM), with no significant difference in activity between the enantiomers (*R*,*R* or *S*,*S* TsDPEN). Non-toxic concentrations of sodium formate potentiate the anticancer activity of the complexes indicating that the complexes might act as transfer hydrogenation catalysts for pyruvate reduction in cells. Lactate dehydrogenase reduces cytosolic pyruvate to l-lactate in cells.^106^ Remarkably, the *R*,*R* enantiomer of the complexes increased the d-lactate concentration in cells when they were co-incubated with sodium formate, suggesting that the catalysts can carry out enantioselective transfer hydrogenation of pyruvate in cells. Since cell survival was not significantly influenced by co-administration of osmium catalysts and formate to non-cancerous cells, pyruvate may be new cellular target for design of the next generation of anticancer drugs.

## Photo-catalytic oxidation of NADH

**Fig. 17:**
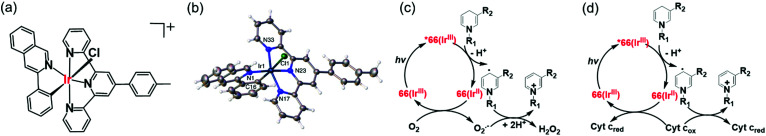
(a) Line structure, and (b) X-ray crystal structure of photocatalyst complex **66**; the counter ion PF_6_^−^ is omitted for clarity. Reproduced from ref. 51 with permission. Catalytic cycle for photo-oxidation of NADH by complex **66**; (c) under normoxia, and (d) under hypoxia in the presence of Fe^3+^–cyt *c* as the terminal electron acceptor.^51^

Photo-catalysis can achieve novel chemical transformations with high yields of products and high reaction specificity.^120–122^ The stable cyclometalated luminescent Ir(iii) catalyst [Ir(ttpy)(pq)Cl]PF_6_ (**66**, Fig. 17a), containing tridentate ttpy = 4′-(*p*-tolyl)-2,2′:6′,2′′-terpyridine, and bidentate pq = 3-phenylisoquinoline ligands, synthesized by treating [Ir(ttpy)Cl_3_] with excess of 3-phenylisoquinoline in glycol under N_2_, can oxidise NADH photocatalytically in cells.^51^ DFT calculations indicated that the *trans* C–Cl isomer found in the crystal structure (Fig. 17b) is significantly more stable than the *cis* C–Cl isomer.^51^

Photo-stable complex **66**, has an extremely high excited-state reduction potential (
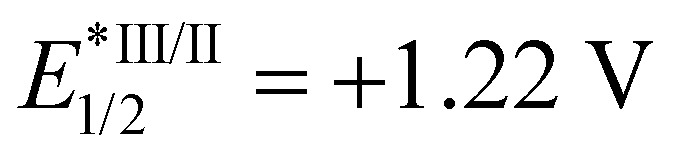
*versus* the saturated calomel electrode, where *E*_1/2_ is the half-wave potential), and photo-catalytically oxidises NADH to NAD^+^*via* NAD˙ radical formation with a high turnover frequency under normoxia. Molecular O_2_ plays an important role in regenerating the active Ir(iii) catalyst from the Ir(ii) state and is converted to the reactive oxygen species H_2_O_2_ (Fig. 17c). In DFT models, the chelated ligands ttpy and pq can π-stack with NADH, positioning the triplet-excited-state hole close to an NADH electron-donor site. Moreover, this complex catalytically reduces cytochrome *c* in the presence of NADH under hypoxic conditions (Fig. 17d).

**Fig. 18:**
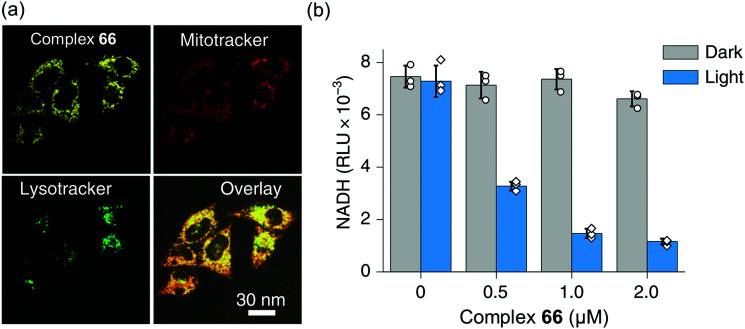
(a) Confocal microscopy images showing mitochondrial localization of the photosensitiser complex **66**. (b) NADH concentration in A549 cells treated with complex **66** with or without photo-irradiation. Reproduced from ref. 51 with permission.

Interestingly, the photosensitizer complex **66** exhibited high immunogenic apoptotic phototoxicity under both normoxia and hypoxia (IC_50_*ca.* 1–8 μM), whilst having low toxicity both in the dark (IC_50_ 17–50 μM) and towards normal MRC-5 human lung fibroblasts and LO2 human hepatocyte cells. Complex **66** localized in mitochondria (Fig. 18a), where both NADH and cytochrome *c* play crucial roles in electron transport, and induced intracellular NADH depletion upon light irradiation (Fig. 18b). In contrast to current photosensitizers, complex **66** generates ROS, decreases the mitochondrial membrane potential, and depletes intracellular NADH under both normoxia and hypoxia. Complex **66** also showed high photocytotoxicity on two-photon red light irradiation (760 nm, 12 J cm^−2^) against A549 multicellular cancer spheroids, a model for solid tumours.

In view of the therapeutic resistance of hypoxic tumours,^123–125^ this catalyst might provide the basis for a new generation of hypoxia-active anticancer agents. Furthermore, its mitochondrial targeting ability has the potential to bypass nucleotide excision repair (NER), one of the factors responsible for acquired drug resistance of Pt chemotherapeutics, as NER is not involved in the repair of mitochondrial damage.^126^

The NAD^+^/NADH redox couple is emerging as the new target for next-generation anticancer drugs.^21–28,31–36,51^ Considering the vital role of these coenzymes in mitochondrial electron transport chain, cell metabolism, enzymatic reactions and several other biochemical pathways, changes of the intracellular NAD^+^/NADH ratio can ultimately lead to cell death.^21–28,31–36,51^ Cancer cells with their disturbed mitochondrial functions are highly sensitive to intracellular redox balance changes, especially those caused by changes of the NAD^+^/NADH ratio.^51^ Thus either intracellular NAD^+^ reduction or NADH oxidation by transfer hydrogenation catalysis might provide a novel way to achieve tumor targeting anticancer activity, and remains to be further investigated.

## In-cell photo-catalytic reduction by flavins

Flavins (FAD or FMN), can be reduced upon light activation in the presence of a sacrificial electron donor such as EDTA.^127,128^ Flavin-coupled systems are widely employed to achieve both oxidation and reduction for numerous organic transformations.^129^ The general mechanism of such reactions involves photoactivation of flavins to an excited state which can extract electrons from a sacrificial electron donor. The photo-generated reducing equivalent can now reduce and activate various molecules, including pro-drugs.

**Fig. 19:**
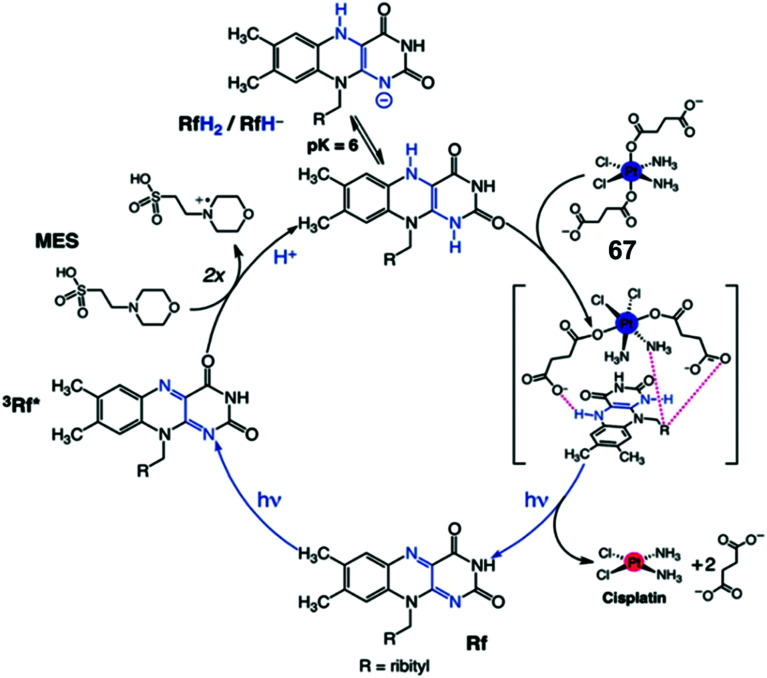
Photo-catalytic cycle for reduction of Pt(iv) prodrug **67** by riboflavin (Rf) upon 460 nm light irradiation. Reproduced from ref. 52; S. Alonso-de Castro *et al*., *Chem. Sci*., 2017, **8**, 4619, published by The Royal Society of Chemistry.

Riboflavin (Rf) can catalyze the photo-reduction of Pt^IV^ prodrugs to active cisplatin under physiologically relevant conditions.^52^ The two-electron-reduced flavin, formed after accepting electrons from a sacrificial electron donor (MES; (2-(*N*-morpholino)ethanesulfonic acid buffer), is the active catalyst (Fig. 19).^52^ The active catalyst appears to form a transient adduct with the Pt^IV^ prodrug and this catalyst–substrate interaction facilitates the transfer of electrons to the Pt^IV^ centre (Fig. 19). Importantly, this chemistry can be translated from reaction flasks to cells. The Rf/**67** pair shows dose-dependent light- (460 nm, 0.36 J cm^−2^) induced anticancer activity against PC-3 (human prostate cancer) cells, with comparable anticancer activity to cisplatin in the dark. All the four components *viz.*, Rf, complex **67**, MES and light are necessary for activity towards PC-3 cells. Cisplatin-like apoptosis was observed as the mode of cell death. Moreover, such a Rf–Pt^IV^ photocatalyst–substrate pair was effective as a photoactive anticancer agent against pancreatic cancer cells.^53^

**Fig. 20:**
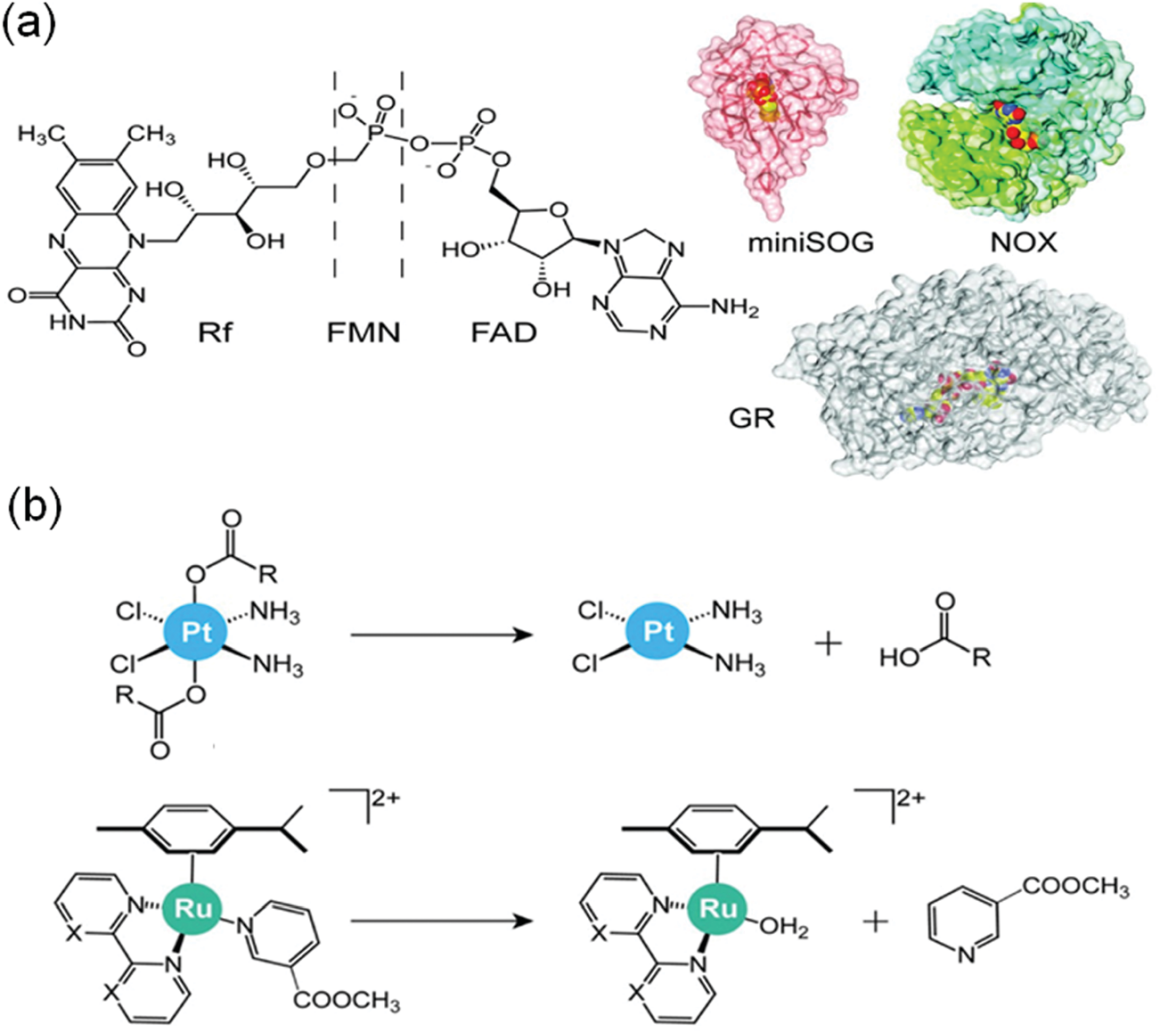
(a) Flavins and flavoproteins for the catalytic photoactivation of anticancer metal complexes and (b) their selected reactions. Reproduced from ref. 20 and 54 with permission.

Interestingly, flavoproteins and flavoenzymes can be used as photocatalysts to convert Pt^IV^ prodrugs to active Pt^II^ complexes by photoreduction as well as Ru^II^ prodrugs to active Ru^II^ active species by release of a monodentate ligand, just like the flavins alone do (Fig. 20).^54^ The flavoprotein mini singlet oxygen generator (miniSOG) and NADH oxidase (NOX) photo-catalytically reduce Pt^IV^ prodrugs in the presence of electron donors (*e.g.* MES, NADH) and irradiation with low doses of visible light (460 nm, 6 mW cm^−2^). Remarkably, NOX, in the presence of NADH as the electron donor, catalyzes Pt^IV^ activation even in the dark indicating that flavoenzymes themselves may activate Pt^IV^ pro-chemotherapeutics using endogenous NADH as the electron source.

## Concluding remarks

Metals are in the active sites of about 40% of all enzymes.^13^ Achieving catalytic reactions similar to those achieved by natural metalloenzymes in cells by synthetic metal-based catalysts is highly challenging on account of the lack of features enjoyed by natural metalloenzymes, including a protein scaffold able to select out substrates from the complicated chemical environment, and a means to shield the active metal centre from inhibitors (poisons). However, even natural metalloenzymes have to cope with some degree of mis-match in substrate selection, and poisoning, which can result in their degradation. Indeed mechanisms to switch the activity of metalloenzymes on and off are vital for the regulation of biochemical pathways. Also not all natural metalloenzyme centres are well shielded by such surrounding scaffolds, *e.g.* Mg^2+^ in ribozymes. Small metal complexes can be viewed as potential metalloenzyme mimics, but equally interesting is the potential for small metal catalysts to carry out unnatural reactions in cells that might lead to new applications in biotechnology and medicine.

Here we have focused on recent advances in the design of metal complexes which catalyse transfer hydrogenation reactions. Such catalysts are widely used on a large scale in the chemicals industry. Examples are chiral tosyldiphenylethylenediamine Ru(ii) (Noyori) catalysts for the reduction of ketones and imines to alcohols and amines using *e.g.* isopropanol as a hydride source. The challenge is to translate such catalysis from non-aqueous solvents into the aqueous media of biological cells.

There is now a range of organometallic complexes in the general class of half-sandwich organometallic Ru(ii), Os(ii) arenes and Rh(iii), Ir(iii) cyclopentadienyl complexes which can catalyse transfer hydrogenation in aqueous media, typically using formate as hydride donor. Formate is relatively nontoxic to cells and is a natural metabolite. Importantly, complexes such as **1–30** can be tailored so as to accept hydride from formate and subsequently reduce NAD(P)^+^, or alternatively for *e.g.* complexes **31–58** to accept hydride from NAD(P)H and generate NAD(P)^+^ and reduce a substrate such as a quinone or O_2_. Moreover, complex **32** can convert NADH into a sequential one electron donor to quinones *via* a reduced Ir(ii) intermediate. The Os(ii) complexes **59–65** have higher catalytic efficiency than their Ru(ii) analogues and are more stable. They can achieve asymmetric hydrogenation in cells and convert pyruvate into unnatural d-lactate. The photocatalyst **66** is stable in the dark, but can extract one electron from NADH on irradiation with visible light both in solution and in cancer cells providing novel impetus in photo-activated cancer drug development with spatiotemporal control over drug activation.

Our review illustrates that metal complex-mediated transfer hydrogenation catalysis can successfully be achieved in cells. Catalytic efficiencies of such catalysts can be tuned with a judicial selection of ligand systems. Such catalysis leads to the reduction of NAD^+^/pyruvate or oxidation of NADH and can potentially create redox imbalance in cells or metabolic disorder – either of which is highly useful to achieve anticancer activity. Moreover, synthetic NADH mimics can also be used for certain bio-catalytic reactions in cells, and are promising for use in biomedical research on drug development. Flavins and flavoproteins can activate cancer prodrugs in a bio-friendly approach for tumour targeting cancer therapy. Thus, in-cell catalysis might provide promising catalytic anticancer drug development strategies, as is evident from the initial *in vitro* data. Moreover such catalysts can also transfer hydride from hydride sources to organic electrophiles such as quinones or aldehydes. This in-cell transfer hydrogenation strategy is expected to be useful in new biotechnologies and novel intracellular bio-conjugation methodology.

These promising in-cell catalyses pave the way for more detailed investigations on small metal catalysts to (i) achieve substrate specificity in cells, (ii) improve the lifetime of the catalysts (towards deactivation after a few cycles), (iii) discover other biocompatible hydride donors, (iv) determine how the distribution and compartmentalization of the catalyst in cells (*e.g.* amongst the cellular organelles and membranes) influences its performance, and importantly (iv) identify applications which can be validated both *in vitro* and *in vivo*.

## Conflicts of interest

There are no conflicts of interest to declare.

## Supplementary Material
